# Six-minute walk distance predicting the risk of mortality in lymphangioleiomyomatosis patients

**DOI:** 10.1186/s13023-026-04194-9

**Published:** 2026-01-10

**Authors:** Luning Yang, Xiaoxin Zhang, Luyi Wang, Chongsheng Cheng, Hanghang Wang, Miaoyan Zhang, Song Liu, Wenshuai Xu, Junya Liu, Jinrong Dai, Shuzhen Meng, Yanli Yang, Shao-Ting Wang, Xinlun Tian, Kai-Feng Xu

**Affiliations:** 1 Department of Pulmonary and Critical Care Medicine, State Key Laboratory of Complex Severe and Rare Diseases, Peking Union Medical College Hospital, Chinese Academy of Medical Sciences & Peking Union Medical College, Beijing, China; 2 Center for Bioinformatics, National Infrastructures for Translational Medicine, Institute of Clinical Medicine, Chinese Academy of Medical Sciences & Peking Union Medical College, Beijing, China; 3 Department of Pulmonary and Critical Care Medicine, Tsinghua University Affiliated Beijing Tsinghua Changgung Hospital, Beijing, China

**Keywords:** Lymphangioleiomyomatosis, 6-minute walk test, Mortality

## Abstract

**Background:**

The study aims to explore the value of the six-minute walk test (6MWT) in assessing the severity and prognosis of patients with lymphangioleiomyomatosis (LAM).

**Methods:**

A retrospective analysis was conducted on 403 LAM patients from the LAM registry study at the Peking Union Medical College Hospital (PUMCH). Survival and progression datasets were constructed. Results: ROC curve analysis established the optimal cut-off value for the six-minute walk distance (6MWD) to predict increased risk of mortality was 425.5 m. The 6MWD low group (6MWD < 425.5 m) exhibited lower SpO₂, higher rates of desaturation, higher post-6MWT Borg dyspnea scale scores, worse pulmonary function indicators (FEV_1_%pred and DLCO%pred), and poorer quality of life assessments (SGRQ) (*P* < 0.001). Furthermore, the 6MWD showed a significant positive correlation with baseline FEV_1_%pred (*P* < 0.001). Multivariate regression analysis indicated that 6MWD, age, desaturation, and post-6MWT Borg dyspnea scale scores were independently correlated with baseline FEV_1_%pred (*P* < 0.001). Progression dataset analysis demonstrated no significant correlation between 6MWT parameters and the annual decline in FEV_1_. Kaplan-Meier survival curves showed a significantly reduced survival probability for patients with 6MWD < 425.5 m, desaturation, or post-6MWT Borg dyspnea scale≥2. Multivariate Cox regression indicated that 6MWD < 425.5 m (HR = 3.759, *P* = 0.0375), FEV_1_%pred < 70% (HR = 12.48, *P* = 0.0045), and sirolimus (HR = 0.1194, *P* < 0.001) were independent factors affecting survival in patients.

**Conclusions:**

6-minute walk distance effectively reflects the physical condition and prognosis and can be utilized as an important tool for clinical assessment in LAM patients.

**Supplementary Information:**

The online version contains supplementary material available at 10.1186/s13023-026-04194-9.

## Introduction

Lymphangioleiomyomatosis (LAM) is a rare and low-grade malignant tumour that occurs almost exclusively in females, particularly those of childbearing age. There are two LAM subtypes, sporadic LAM and tuberous sclerosis complex (TSC) associated LAM [1]. Those two types of LAM are caused by the somatic and germline *TSC2* gene mutation respectively [2, 3]. The mutant TSC genes result in downstream activation of the mammalian target of rapamycin (mTOR) signalling pathway, which leads to abnormal cell growth and proliferation. Such aberrantly proliferating cells can induce diffuse cystic lesions in the lungs, leading to dyspnea, decreased oxygen saturation, and hemoptysis [1]. They may also result in complications such as pneumothorax, chylothorax, and pulmonary hypertension [1]. Other clinical manifestations include enlarged lymph nodes in the abdomen and pelvis, as well as renal angiomyolipomas [1].

In LAM patients, pulmonary function decline may precede the appearance of any symptoms. The forced expiratory volume in 1 s (FEV_1_) of LAM patients declines at a rate of 89.53 ml decline per year on average [4, 5], approximately three times that of normal people. As the disease progresses, patients may develop hypoxemia and respiratory failure, which severely impact their quality of life. Patients may need a lung transplant to extend their lives at this point. Sirolimus, an mTOR inhibitor, has now proven to have a significant effect on LAM [6]. Current guidelines recommend the use of sirolimus when FEV_1_ is under < 70% of the predicted value or the decline of FEV_1_ is more than 90 ml per year [7].

Therefore, regular follow-up to assess the severity and the progression of disease is very important for patients. Pulmonary function testing is commonly used in evaluation of severity and progression. It has been reported that patients whose FEV_1_ declines faster are more likely to have a poorer prognosis and worse quality of life [1, 5]. Serum VEGF-D can also be used to evaluate the severity of LAM. Existing studies have shown that elevated baseline VEGF-D levels are associated with reduced DLCO and worse quality of life [5, 8]. Imaging examinations can also be used for assessment. Patients with lung CT grading as level II or level III usually have higher levels of VEGF-D and are at a higher risk of pneumothorax [9, 10].

However, each of these assessment methods has its limitations. Pulmonary function tests require not only specialized equipment but also good cooperation of the patient to obtain a reliable result. Patients with LAM may refuse pulmonary function testing due to the difficulty in cooperating or concerns about the risk of pneumothorax. Also, LAM patients with pneumothorax, chylothorax, or significant dyspnea are unable to undergo a pulmonary function test. As for the VEGF-D test, it is currently available in only a small number of hospitals, and its result could be affected because of improper transportation or storage. Although CT scans can visually show the extent of cystic lesions, it is usually difficult to quantify the annual changes of lung lesions, making it challenging to assess progression by CT images.

The 6-minute walk test (6MWT) is one of the commonly used methods for assessing LAM. It is widely used to evaluate chronic obstructive pulmonary disease and a range of other diseases [11]. It is a simple, safe, and easily implemented test of exercise ability. Patients are asked to walk as quick as possible on a flat surface for 6 min and the walking distance will be record, called the 6-minute walk distance (6WMD). Other indicators that would be recorded include the patient’s peripheral oxygen saturation (SpO₂), the Borg dyspnea scale and any self-reported discomfort, such as dyspnea, dizziness, or headache. Compared with pulmonary function tests, the 6MWT does not require complicated equipment or training. Therefore, it can be easily conducted. The 6MWT is also capable of capturing the extrapulmonary manifestations which often accompany chronic respiratory diseases, such as cardiovascular disease, frailty, or sarcopenia.

For the mentioned reasons, 6MWT are wildly used in evaluating patients in clinical practice. It was first developed to evaluate exercise capacity of patients with chronic obstructive pulmonary disease (COPD). Research reports have shown that the 6MWT is a good predictor of mortality risk of COPD [12–14]. It can also be used to assess the risk of hospitalization or acute exacerbations [15]. 6MWT is also recommended as a tool to investigate patients’ exercise capacity in idiopathic pulmonary arterial hypertension (PAH) [16]. An investigation has indicated that the 6MWD can predict their 1-year mortality and survival rates [17]. Many other research also proved that 6MWD and desaturation are significant indicators of predicting outcomes for PAH patients [18–20]. Likewise, the 6MWT has been explored for prognostic prediction in various diseases, including idiopathic pulmonary fibrosis [21], lung transplantation [22, 23], and heart failure [24]. The current guidelines also recommend 6MWT as a method for assessing exercise capacity in patients with LAM [25]. The 6MWD has also been used as an outcome measure in several LAM-related studies [6, 26]. Currently, there is only a few studies exploring the efficacy of the 6MWT in assessing disease severity and prognosis in LAM patients. Baldi et al. revealed a significant correlation between the extent of pulmonary cysts and the degree of desaturation after 6MWT in LAM patients [27].

Two studies from Peking Union Medical College Hospital (PUMCH) found that sirolimus treatment can effectively improve patients’ 6MWD, as well as enhance their oxygen saturation levels and pulmonary function [5, 28]. These studies suggest that the 6MWT may have the potential to be an effective tool to assess the severity and progression of LAM.

This study, based on the LAM cohort in PUMCH, aims to investigate the ability of 6MWT to evaluate LAM severity and progression. Hopefully, it will provide a more convenient method for evaluating LAM patients, enable the patients and physicians to learn more about the physical condition of patients and implement appropriate interventions.

## Methods

### Study population

**Fig. 1:**
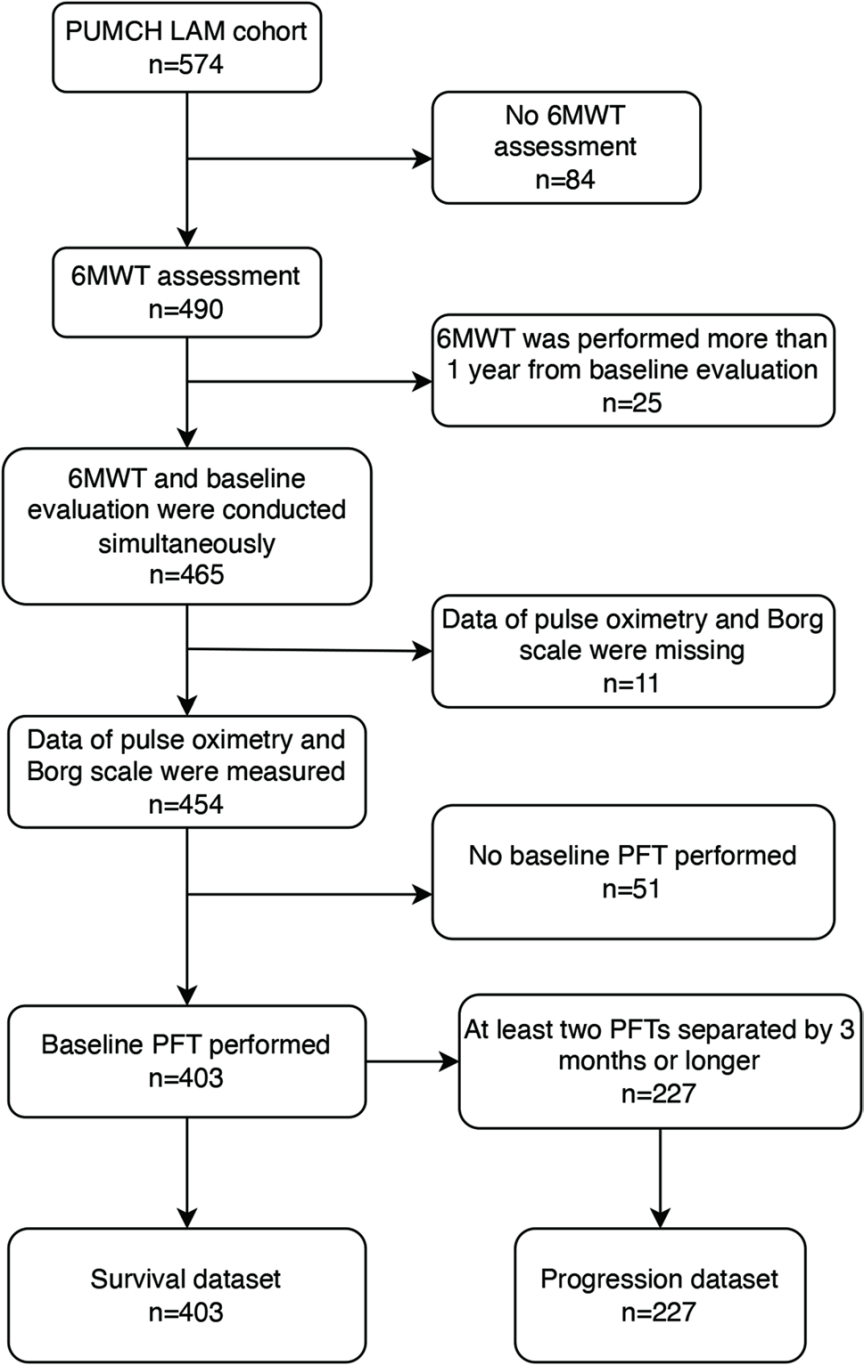
Flowchart of the study population. PUMCH, Peking Union Medical College Hospital; LAM, lymphangioleiomyomatosis; 6MWT, 6-minute walk test; PFT, pulmonary function test

This is a single-center retrospective study. Patients were enrolled from the LAM registry study conducted at PUMCH, and progression datasets and survival datasets were constructed as described previously [5]. Briefly, as shown in Fig. 1, patients with a diagnosis of definite LAM were enrolled, and those with no baseline 6MWT or PFTs assessment or incomplete data were excluded. For the survival dataset, all patients with complete baseline evaluation were included, and the follow-up ended at death, lung transplant, lost to follow-up, or December 31st, 2019, whichever came first. For the progression dataset, at least two pulmonary function tests separated by at least three months were required. The study protocol was approved by the PUMCH Institutional Review Board (approval numbers: S-379 and JS-1323) in accordance with the Declaration of Helsinki, the study was registered on ClinicalTrials.gov (NCT03193892, January 1, 2017), and informed consent was obtained in all patients.

### Clinical evaluation

The following information was gathered: age, menopause status, history of pneumothorax, chylothorax, renal angiomyolipomas, retroperitoneal lymphangioleiomyomas, and TSC. Sirolimus exposure at baseline was defined as treatment with sirolimus for at least three months before baseline evaluation. Sirolimus treatment thereafter was defined as treatment for at least three months before the end of follow-up. Pulmonary function tests [29, 30], arterial blood gas at room air, and St. George’s Respiratory Questionnaire (SGRQ) [31] were evaluated as previously described [5] at baseline and each follow-up. A high-resolution CT scan of the lung was performed, and the severity was determined based on the extent of cystic involvement: Grade 1 indicated less than 1/3 of the lung, while Grade 2 between 1/3 and 2/3, and Grade 3 more than 2/3 [32]. Serum vascular endothelial growth factor-D (VEGF-D) levels were measured by the enzyme-linked immunosorbent assay (ELISA) kit (R&D Systems, Minneapolis, MN, USA, #DVED00) [10]. VEGF-D was measured simultaneously as the clinical evaluation was performed at baseline.

### Six-minute walk test (6MWT)

The 6-minute walk tests were evaluated by the ATS Guidelines [33]. Before the test, the patient should rest for at least five minutes, and pre-test vital signs and SpO_2_ were collected. The test was performed in a flat, straight, and hard-surfaced corridor, and the one-way length of the walking route was thirty meters. During the test, a physician provided a series of standard instructions to the patient every minute. After the test, a physician calculated the total walking distance (6MWD), measured the post-test vital signs and SpO_2_, assessed the modified Borg dyspnea scale [34], and documented any unexpected situation that happened. If patients were under long-term oxygen therapy, they would be asked to take oxygen during 6MWT [33]. However, those patients always refuse 6MWT instead of completing the test with oxygen supply. Few patients finished the test with oxygen supplementation. The test would be immediately stop if patients felt any intolerable discomfort including chest pain, dyspnea, exhaustion or pale [33]. 6MWT was performed at baseline. As commonly used in COPD patients, desaturation during 6MWT was defined as SpO_2_ decline more than 4% or less than 90% post-test [12], and exercise-induced desaturation (EID) was defined as SpO_2_ decline more than 4% and less than 90% post-test [35].

### Statistical analysis

Continuous variables were presented as mean ± SD for normally distributed variables or median with interquartile range (IQR, 25th-75th percentiles) for those with non-normal distribution, while categorical variables were reported as numbers with percentiles. Student t-tests were used to compare continuous variables with normal distribution, and Wilcoxon rank-sum tests for non-normal distribution. Chi-square tests or Fisher’s exact test were used to assess differences in categorical variables. Correlations between 6MWD and FEV_1_%pred were estimated by Spearman. A receiver operating characteristic (ROC) analysis with mortality was used to determine the best cut-off point for the 6MWD. Annual decrease rate of FEV_1_ was calculated with a mixed-effects model with a random intercept and random slope. Baseline FEV_1_ was set as the fixed effect and the patients age and time of follow-up was set as random effects. Multivariable generalized linear regression was used to determine parameters associated with baseline FEV_1_%pred and annual decrease of FEV_1_. Kaplan-Meier curves were drawn to display the differences in mortality. Factors related to death/lung transplant were identified by a multifactorial Cox regression model. A two-tailed P value less than 0.05 was considered statistically significant. Data were analysed using R version 4.4.3 (The R Foundation for Statistical Computing).

## Results

**Fig. 2:**
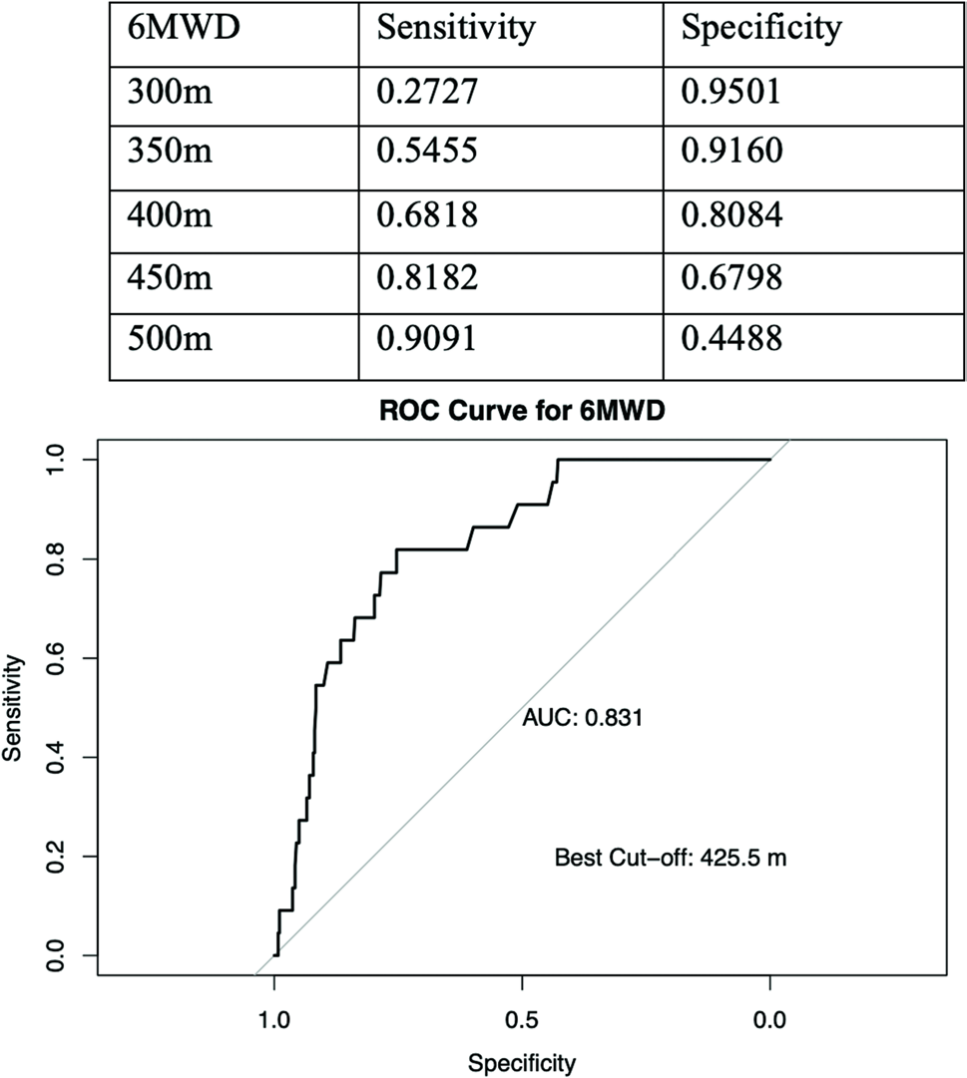
The receiver operating characteristic curve of 6MWD threshol. The table presents sensitivity and specificity at various 6MWD thresholds. The ROC curve illustrates the trade-off between sensitivity and specificity across different threshold values

Demographic data of the patients enrolled were summarized in Table 1. A total of 403 LAM patients were included in the study, with 381 surviving, 21 deceased and 1 recipient of lung transplant (divided into deceased group in the following analysis). Among the 21 deaths, 18 resulted from complications of LAM, 1 was caused by hepatocellular carcinoma, and the underlying causes of death were unknown in two cases. The analysis of baseline data revealed a significant difference in the 6-minute walk distance (6MWD) between surviving (485, 426–533) and deceased (347, 300.25-405.12) patients (*P* < 0.001), with deceased patients showing a shorter 6MWD. Furthermore, the pre-6MWT blood oxygen saturation (SpO_2_), post-6MWT blood oxygen saturation (SpO_2_), presence of desaturation and exercise-induced desaturation also showed significant differences between the two groups (*P* = 0.002, 0.005, 0.048, and < 0.001, respectively). Additionally, the post-6MWT Borg dyspnea scale significantly increased in deceased patients (*P* < 0.001). Further analysis of the receiver operating characteristic (ROC) curve for the 6MWD threshold revealed that the cut-off value of 425.5 m for 6MWD provided the best prediction of mortality (Fig. 2).

**Table 1:** Baseline characteristics of the patients in the survival dataset

Variables	Total (*n* = 403)	Survivors (*n* = 381)	Deceased (*n* = 22)	*P*
6MWD, m	480 (410.5, 530)	485 (426, 533)	347 (300.25, 405.12)	< 0.001^‡^
Pre-6MWT SpO_2_, %	98 (96, 99)	98 (96, 99)	95.5 (94, 97.75)	0.002^‡^
Post-6MWT SpO_2_, %	97 (90, 99)	97 (91, 99)	89.5 (81.25, 96.75)	0.005^‡^
Desaturation	133 (33)	121 (32)	12 (55)	0.048^§^
Exercise-induced Desaturation	54 (13)	43 (11)	11 (50)	< 0.001^§^
Post-6MWT Borg dyspnea scale	1 (0, 2)	1 (0, 2)	3 (2, 4.75)	< 0.001^‡^
Age, y	39 (31, 46)	39 (31, 46)	39 (30.25, 44)	0.619^‡^
Menopause	61 (15)	61 (16)	0 (0)	0.034^§^
History of smoking	6 (1)	6 (2)	0 (0)	1^§^
History of pneumothorax	128 (32)	119 (31)	9 (41)	0.476^§^
History of chylothorax	58 (14)	56 (15)	2 (9)	0.754^§^
Renal AMLs	138 (34)	134 (35)	4 (18)	0.161^§^
Retroperitoneal LAMs	80 (20)	78 (20)	2 (9)	0.274^§^
TSC	34 (8)	34 (9)	0 (0)	0.24^§^
VEGF-D, pg/ml	2014.39 (1067.06, 3351.84)	1798.55 (1038.72, 3340.31)	3075.6 (2179.81, 3428.3)	0.027^‡^
CT severity grade				0.012^*^
1	65 (16)	65 (17)	0 (0)	
2	64 (16)	63 (17)	1 (5)	
3	274 (68)	253 (66)	21 (95)	
PaO_2_, mmHg	73.7 (50.3, 89.75)	75.3 (52, 90.1)	41.25 (28, 61.17)	< 0.001^‡^
FEV_1_%pred	90.8 (77.2, 102.75)	91.8 (78.7, 103.2)	71.5 (60.2, 85.77)	< 0.001^‡^
FVC%pred	69.6 (51.52, 79.28)	70.94 (53.84, 79.59)	48.54 (40.57, 52.4)	< 0.001^‡^
FEV_1_/FVC	123.9 (107.27, 160.43)	122.2 (106.2, 158.25)	172.5 (154.6, 223.5)	< 0.001^‡^
RV%pred	101 (91.57, 113.03)	101 (91.2, 112.9)	108 (98.8, 127.4)	0.071^‡^
DLCO%pred	49.39 (33.8, 68.44)	50.4 (34.8, 68.7)	31.8 (23.6, 43.65)	< 0.001^‡^
SGRQ domain score	36 (19, 53)	33.5 (18, 52)	64.5 (50, 74)	< 0.001^‡^
Sirolimus treatments	279 (69)	268 (70)	11 (50)	0.076^§^

6MWD, six-minute walk distance; 6MWT, 6-minute walk test; AMLs, angiomyolipomas; retroperitoneal LAMs, retroperitoneal lymphangioleiomyomas; TSC, tuberous sclerosis complex; VEGF-D, vascular endothelial growth factor D; SGRQ, St. George’s Respiratory Questionnaire. ‡. Wilcoxon rank-sum test; §. Chi-square test; *. Fisher’s exact test. Data are presented as No. (%) or median (interquartile range)

**Fig. 3:**
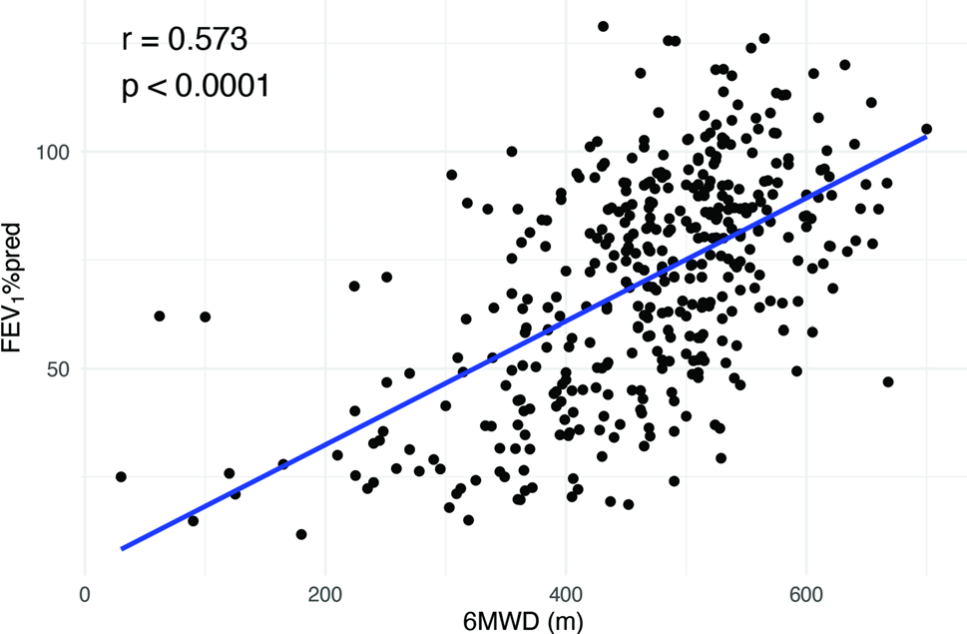
Relationship between 6MWD and FEV_1_%pred in LAM. 6MWD, six-minute walk distance; LAM, lymphangioleiomyomatosis

Patients were categorized into two groups, 6MWD high (6MWD≥425.5 m, *n* = 291) and 6MWD low (6MWD<425.5 m, *n* = 112). As summarized in Table 2, results showed that patients in the 6MWD low group had significantly lower pre- and post-6MWT SpO_2_, higher rate for desaturation, and higher post-test Borg dyspnea scale compared to the 6MWD high group (*P* < 0.001). Additionally, the 6MWD low group exhibited worse pulmonary function indicators such as FEV_1_%pred, DLCO%pred, and quality of life assessments (SGRQ) (*P* < 0.001). The study also found a significant positive correlation between 6MWD and FEV_1_%pred (Fig. 3). Through multiple generalized linear regression analyses, factors such as 6MWD, age, desaturation, and post-6MWT Borg dyspnea scale scores≥2 and CT severity grade were independently associated with baseline FEV_1_%pred (*P* < 0.001) (Table 3).

**Table 2:** Comparison of characteristics according to 6MWD in LAM patients

Variables	Total (*n* = 403)	6MWD High (*n* = 291)	6MWD Low (*n* = 112)	*P*
6MWD, m	480 (410.5, 530)	510 (470, 545)	362.5 (308, 396)	< 0.001^‡^
Pre-6MWT SpO_2_, %	98 (96, 99)	98 (96, 99)	96 (93, 98)	< 0.001^‡^
Post-6MWT SpO_2_, %	97 (90, 99)	97 (93, 99)	92 (84.5, 98)	< 0.001^‡^
Desaturation	133 (33)	78 (27)	55 (49)	< 0.001^§^
Exercise-induced Desaturation	54 (13)	22 (8)	32 (29)	< 0.001^§^
Post-6MWT Borg dyspnea scale	1 (0, 2)	0.5 (0, 2)	3 (1, 4)	< 0.001^‡^
Age, y	39 (31, 46)	38 (32, 45)	40 (29, 47.25)	0.293^‡^
History of pneumothorax	128 (32)	89 (31)	39 (35)	0.485^§^
History of chylothorax	58 (14)	36 (12)	22 (20)	0.088^§^
Renal AMLs	138 (34)	106 (36)	32 (29)	0.17^§^
Retroperitoneal LAMs	80 (20)	64 (22)	16 (14)	0.11^§^
TSC	34 (8)	29 (10)	5 (4)	0.114^§^
VEGF-D, pg/ml	2014.39 (1067.06, 3351.84)	1669.75 (956.37, 3244.42)	2652.15 (1561.6, 3443.12)	< 0.001^‡^
CT severity grade				< 0.001^*^
I	65 (16)	59 (20)	6 (5)	
II	64 (16)	58 (20)	6 (5)	
III	274 (68)	174 (60)	100 (89)	
PaO_2_, mmHg	79.91 ± 15.22	84.16 ± 13.94	68.67 ± 12.56	< 0.001^‡^
FEV_1_%pred	73.7 (50.3, 89.75)	81 (64.15, 92.7)	44.8 (29.75, 63.92)	< 0.001^‡^
DLCO%pred	49.39 (33.8, 68.44)	55 (41.58, 71.2)	31.92 (22.12, 48.21)	< 0.001^‡^
SGRQ domain score	36 (19, 53)	28 (16, 43)	57 (46.5, 71)	< 0.001^‡^
Sirolimus exposure before baseline	18 (4)	12 (4)	6 (5)	0.789^§^
Death	22 (5)	5 (2)	17 (15)	< 0.001^§^

6MWD, six-minute walk distance; 6MWT, 6-minute walk test; retroperitoneal LAMs, retroperitoneal lymphangioleiomyomas; AMLs, angiomyolipomas; TSC, tuberous sclerosis complex; VEGF-D, vascular endothelial growth factor D; SGRQ, St. George’s Respiratory Questionnaire. ‡. Wilcoxon rank-sum test; §. Chi-square test; *. Fisher’s exact test. Data are presented as No. (%) or median (interquartile range)

**Table 3:** Multivariate analysis of parameters associated with baseline FEV_1_%pred in LAM

Variable	Estimate	95% CI	*P*
Intercept	42.85	27.88 to 57.82	< 0.001
Age	0.4024	0.2192 to 0.5856	< 0.001
6MWD	0.09826	0.07898 to 0.1175	< 0.001
Desaturation	-7.626	-11.71 to -3.542	< 0.001
Post 6MWT Borg dyspnea scale≥2	-7.730	-11.77 to -3.69	< 0.001
VEGF-D	0.00004903	-0.000768 to 0.000866	0.9065
CT severity grade	-11.27	-13.83 to -8.710	< 0.001
Sirolimus treatment	3.917	-4.557 to 12.39	0.3655

LAM, lymphangioleiomyomatosis; 6MWD, six-minute walk distance; 6MWT, 6-minute walk test; VEGF-D, vascular endothelial growth factor D

To analyse the association of 6MWT parameters and disease progression, we included the progression dataset where all patients had at least two pulmonary function tests separated by three months or longer. Patients were divided into stable and progressive groups based on whether their annual decline in FEV_1_ was greater than 30 ml (sTable 1). The cut-off value of 30 ml/year was chosen in this study because studies have shown that the average annual FEV decline rate in healthy women is approximately 30ml [36]. The results showed that there were no statistically significant differences between the two groups regarding 6MWD (*P* = 0.061), pre-6MWT SpO_2_, post-6MWT SpO_2_, desaturation or exercise-induced desaturation, but significant differences were observed in the prevalence of renal AMLs, TSC, CT severity grade, PaO_2_, FEV_1_/FVC, DLCO%pred, sirolimus treatment and death (*P* = 0.03, 0.003, 0.011, 0.002, 0.01, 0.019, 0.002 and 0.007, respectively). Multiple generalized linear regression analyses (sTable 2) indicated that CT severity grading III, FEV_1_%pred < 70%, and sirolimus treatment were correlated to disease progression (*P*<0.0001, 0.0006, and <0.0001, respectively). A trend toward disease progression was observed in patients with a 6-minute walk distance (6MWD) of < 425.5 m, though this trend did not reach statistical significance.

**Fig. 4:**
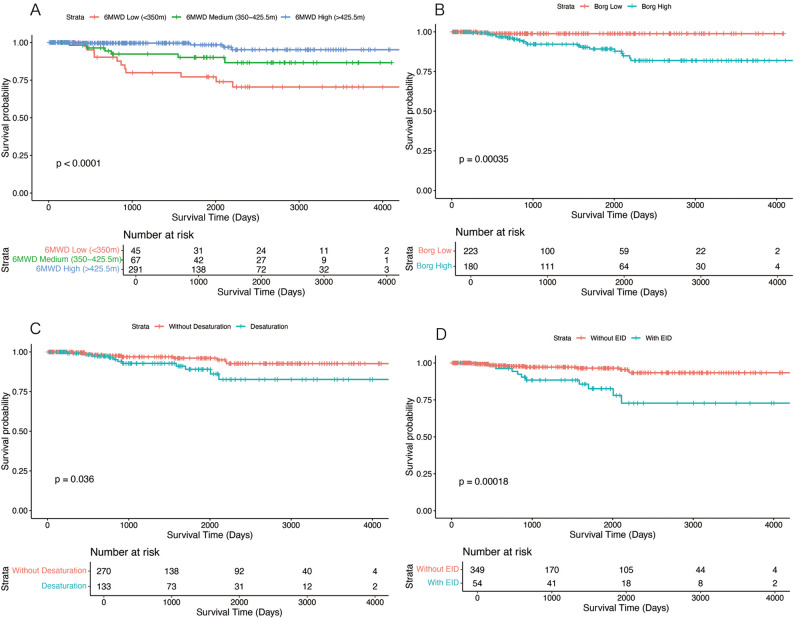
Kaplan-Meier survival curves in patients with different 6MWD (**A**), Borg dyspnea scale (**B**), desaturation (**C**) and exercise-induced desaturation (**D**). 6MWD, six-minute walk distance; 6MWD high, 6MWD≥425.5 m; 6MWD medium, 6MWD 350–425.5 m; 6MWD low, 6MWD < 350 m; Borg low, post-6MWT Borg dyspnea scale < 2; Borg high, post-6MWT Borg dyspnea scale≥2; EID, exercise-induced desaturation

The survival dataset was used to explore the relationship between 6MWT parameters and prognosis (Fig. 4). Kaplan-Meier survival curves showed that survival probability was significantly lower in patients with 6MWD < 425.5 m (Fig. 4A), post 6MWT Borg dyspnea scale≥2 (Fig. 4B), desaturation (Fig. 4C) or exercise-induced desaturation (Fig. 4D). Multifactorial Cox regression showed that 6MWD < 425.5 m (HR = 3.759, 95% CI 1.080–13.08, *P* = 0.0375), FEV_1_%pred < 70% (HR = 12.48, 95% CI 2.184–71.36, *P* = 0.0045), and not using sirolimus (HR = 0.1194, 95% CI 0.045–0.316, *P* < 0.001) are independent risk factors affecting patient survival (Table 4). To test the robustness of the association between 6MWD and survival, a sensitivity test in a validation cohort was performed. The new cohort was constructed by revising the inclusion/exclusion criteria. In the new cohort, it was no longer required for patients to test pulmonary function test and 6MWT simultaneously. Patients with missing data of Borg scale and pulse oximetry were not excluded. Applying these revised inclusion/exclusion criteria, we identified 478 patients, of whom 24 reached the endpoint (death or lung transplant). Cox proportional-hazards regression was retested. The findings confirmed that a 6MWD<425.5 m remained significantly associated with the death and lung transplant (HR 4.74, 95% CI 1.41–15.97, *P* = 0.012), in line with our primary conclusion.

**Table 4:** Multivariable Cox analysis of risk factors for death in LAM

Variables	Hazard Ratio	95% CI	*P*
Age	0.9855	0.943–1.030	0.5161
6MWD < 425.5 m	3.759	1.080–13.08	0.0375
VEGF-D≥800pg/ml	0.2534	0.044–1.463	0.1245
FEV_1_%pred < 70%	12.48	2.183–71.36	0.0045
Sirolimus treatment	0.1194	0.045–0.316	< 0.001

LAM, lymphangioleiomyomatosis; 6MWD, six-minute walk distance; 6MWT, 6-minute walk test; VEGF-D, vascular endothelial growth factor D

## Discussion

This study explored the relationship between 6MWT parameters and clinical manifestations and prognosis in a cohort of 403 patients with LAM. The results showed a significant difference in the 6MWD between the surviving and deceased or lung transplant patients. The best predictive threshold for 6MWD was determined by ROC curve analysis to be 425.5 m. After further dividing the patients into the 6MWD high and low groups, it was found that the patients in the 6MWD low group not only had lower oxygen saturation (SpO₂) before and after 6MWT, higher post-6MWT Borg dyspnea scale, but also had worse pulmonary function indicators (such as FEV_1_%pred, DLCO%pred) and quality of life assessment (SGRQ), which confirmed that patients with lower 6MWD had more severe disease. In addition, the Kaplan-Meier survival curve showed a significantly reduced survival probability in patients with 6MWD < 425.5 m, and multivariate Cox regression analysis showed 6MWD < 425.5 m is an independent risk factor for patient survival, further highlighting the important value of 6MWD in assessing the prognosis of patients with LAM. In addition to the 6MWD, this study also focused on other parameters in the course of the 6MWT, such as oxygen saturation before and after the test, whether desaturation occurred, and the post-6MWT Borg dyspnea scale. The study found that the deceased or lung transplant patients had lower pre-6MWT or post-6MWT SpO_2_, and a higher incidence of desaturation and higher post-6MWT Borg dyspnea scale score. In the analysis of disease progression, there was no significant association determined in the parameters of 6MWT and the annual decrease of FEV_1_.

Consistent with the results reported by Diesler et al. [37], this study found that the 6-minute walk distance (6MWD) in patients with LAM was associated with several pulmonary function indicators, including FEV_1_%pred, FVC%pred, and DLCO%pred. This indicates that 6MWD can reflect the pulmonary function status of patients at baseline. The 6MWT is a simple, economical, and safe experimental method that can reflect the functional status of patients in multiple dimensions. It is also applicable to patients who have contraindications for pulmonary function tests, such as those with chylothorax or pneumothorax or risk for pneumothorax. Furthermore, 6MWT has been widely applied and has demonstrated good effects in patients with chronic obstructive pulmonary disease (COPD), interstitial lung diseases (ILD), and pulmonary hypertension, further proving its efficacy and practicality in the assessment of respiratory system diseases [12–20].

In LAM patients, we showed that the baseline 6-minute walk distance (6MWD) was associated with baseline FEV_1_%pred and mortality, but no significant correlation was found with annual decline in FEV_1_. Pulmonary function test is an indirect indicator reflecting the destruction of lung structure (such as the progression of cysts), while 6MWD reflects overall cardiopulmonary functional status. The 6MWD is associated with mortality, potentially because a low 6MWD reflects more severe systemic impairment (such as pulmonary hypertension and insufficient cardiopulmonary reserve). But we showed that baseline 6MWD cannot predict a future decline of pulmonary function. We need to take it with caution when using the rate of decline in FEV_1_ to assess total disease progression in LAM, as evaluating only the progression of lung lesions and overlooking the others may have limitations.

A recently published research [38] showed that untreated LAM patients who developed exercise-induced desaturation (EID) had a higher risk of accelerated FEV_1_ loss. In their study, EID was defined as a minimum exercise-related oxygen saturation (SpO_2_) of < 88%. Follow-up time was 65.2 months. In the present study, exercise-induced desaturation (EID) was defined as SpO_2_ decline more than 4% and less than 90% post-test. Desaturation was defined as SpO_2_ decline more than 4% or less than 90% post-tests as commonly used in COPD. And our medium follow-up time was 16.8 months. We did not observe the same tread for EID or desaturation. Although EID and desaturation is associated with death/transplant showed by Kaplan-Meier curve, it is not associated with annual decline of FEV_1_ This difference may due to various EID definition, baseline severity and follow-up time in different cohorts.

In our study, 6MWD < 425.5 m, FEV_1_%pred < 70% and no treatment of sirolimus were variables significantly associated with worse survival [5]. In the study published previous by our group, the significant variables associated with worse survival were VEGF-D≥2000pg/ml, FEV_1_%pred < 70%, SGRQ symptoms domain score≥50 and no sirolimus use. The difference between the two studies may be due to different inclusion/exclusion criteria, sample size, and statistical methods. Our study required patients to have complete baseline 6MWT which may exclude some patients with severe disease (unable to complete 6MWT). In our study, the survival dataset included 403 patients (22 died/lung transplanted) whilst previous study included 574 patients (41 deaths/lung transplanted). In this study, we used a single-variable and multiple-variable Cox regression, while in the previous study, a stepwise Cox proportional hazards analysis was used. These may lead to different results from the two studies.

The advantage of this study is that we systematically analysed the relationship between 6MWT parameters and prognosis in a large cohort of LAM patients, providing valuable reference data for clinical practice. However, the study does have some limitations. Firstly, as a single-center retrospective study, there may be selection bias and information bias, which could affect the external validity of the results. Secondly, the study used the absolute value of 6MWD rather than the percentage of the expected 6MWD for patients, which may influence the comparison of exercise capacity among patients with different BMIs and ages. Thirdly, this study only explored the relationship between the baseline 6-minute walk test and clinical manifestations, and did not investigate the longitudinal changes in the 6-minute walk test parameters during follow-up. Studies [39] have shown that in patients with LAM, the average annual decline in 6-minute walk distance before treatment with sirolimus is 21 ± 6 m, while after sirolimus treatment, 6MWD can increase by 15 ± 3 m. Therefore, future studies could explore the changes in the 6MWT parameters longitudinally before and after treatment, as well as how these changes correlate with pulmonary function test parameters and clinical outcomes.

In conclusion, this study found that a 6MWD of 425.5 m was the optimal cut-off for predicting mortality risk. Further analysis found that 6MWD was positively correlated with baseline FEV_1_, DLCO and health-related quality of life. However, the 6MWT parameters failed to predict the future decline of lung function. This study showed that 6MWT generated valuable parameters to predict the prognosis of LAM patients, serving as an important tool for clinical assessment. In clinical practice, integrating information from 6MWT parameters, pulmonary function indicators, CT imaging severity, VEGF-D and treatment conditions can facilitate a more comprehensive evaluation of the condition and prognosis of LAM patients, thereby providing stronger support for individualized treatment decisions.

## Supplementary Information

Below is the link to the electronic supplementary material.

Supplementary Material 1

## Data Availability

The datasets generated during and analyzed during the current study are available from the corresponding author on reasonable request.
