# Supplementary material for: Six-minute walk distance predicting the risk of mortality in lymphangioleiomyomatosis patients
Source: Orphanet J Rare Dis. 2026 Jan 10;21:47. doi: 10.1186/s13023-026-04194-9 (PMC12882533; doi:10.1186/s13023-026-04194-9)
Supplement: Supplementary file 1 — Supplementary Material 1 [file 13023_2026_4194_MOESM1_ESM.docx]

**Six-minute Walk Distance Predicting the Risk of Mortality in Lymphangioleiomyomatosis Patients**

Luning Yang^1#^, Xiaoxin Zhang^1#^, Luyi Wang^1^, Chongsheng Cheng^1^, Hanghang Wang^1^, Miaoyan Zhang^1^, Song Liu^2^, Wenshuai Xu^1,3^, Junya Liu^1^, Jinrong Dai^1^, Shuzhen Meng^1^, Yanli Yang^1^, Shao-Ting Wang^1^, Xinlun Tian^1^ and Kai-Feng Xu^1*^

sTable 1 Comparison of characteristics between stable and progression groups in LAM patients

| Variables | Total (n = 227) | Progression group  (n = 108) | Stable group  (n = 119) | *P* |
| --- | --- | --- | --- | --- |
| Follow up duration, M | 16.8 (12.1-28.9) | 18.2 (12.3-34.1) | 16.1 (12.1-27.3) | 0.2758^‡^ |
| 6MWD, m | 500 (432.75, 545) | 482.5 (404.38, 539) | 510 (452.5, 545) | 0.061^‡^ |
| Pre-6MWT SpO_2_, % | 98 (96, 99) | 98 (96, 99) | 98 (96, 99) | 0.107^‡^ |
| Post-6MWT SpO_2_, % | 97 (90.5, 99) | 96.5 (90, 98.25) | 97 (92, 99) | 0.132^‡^ |
| Desaturation | 78 (34) | 41 (38) | 37 (31) | 0.343^§^ |
| Exercise-induced Desaturation | 29 (13) | 17 (16) | 12 (10) | 0.282^§^ |
| Post-6MWT Borg dyspnea scale | 1 (0, 2) | 1 (0, 3) | 0.5 (0, 2) | 0.146^‡^ |
| Age, y | 39.37 ± 9.67 | 40.57 ± 9.91 | 38.29 ± 9.36 | 0.076^‡^ |
| Pneumothorax | 43 (19) | 17 (16) | 26 (22) | 0.316^§^ |
| Chylothorax | 15 (7) | 8 (7) | 7 (6) | 0.846^§^ |
| Renal AMLs | 71 (31) | 23 (21) | 48 (40) | 0.003^§^ |
| Retroperitoneal LAMs | 48 (21) | 22 (20) | 26 (22) | 0.913^§^ |
| TSC | 21 (9) | 3 (3) | 18 (15) | 0.003^§^ |
| VEGF-D, pg/ml | 2007.17 (1070.48, 3318.52) | 2060.01 (1170.67, 3309.71) | 1664.62 (1011.75, 3333.91) | 0.288^‡^ |
| CT severity grade |  |  |  | 0.011 ^*^ |
| I | 35 (15) | 10 (9) | 25 (21) |  |
| II | 41 (18) | 16 (15) | 25 (21) |  |
| III | 151 (67) | 82 (76) | 69 (58) |  |
| PaO_2_, mmHg | 81.56 ± 15.17 | 78.21 ± 14.57 | 84.58 ± 15.13 | 0.002^‡^ |
| FEV_1_%pred | 74.8 (52.25, 92.15) | 73.45 (49.5, 92.32) | 75.9 (57.75, 90.2) | 0.551^‡^ |
| FVC%pred | 91.84 ± 19.32 | 92.61 ± 20.41 | 91.15 ± 18.33 | 0.573^‡^ |
| FEV_1_/FVC | 70.99 (53.62, 79.46) | 67.97 (50.74, 77.25) | 74.56 (58.58, 82.34) | 0.01^‡^ |
| RV%pred | 119.2 (104.9, 156.85) | 125 (108, 161.15) | 115.6 (103.2, 149.1) | 0.104^‡^ |
| TLC%pred | 100.55 (91, 112.9) | 102 (93.55, 112.9) | 97.3 (89.7, 112.1) | 0.044^‡^ |
| DLCO%pred | 52.24 (35.8, 71.43) | 46.7 (34.3, 66.09) | 58.3 (41.15, 79.8) | 0.019^‡^ |
| SGRQ domain score | 33 (17, 52) | 39 (20, 56) | 30 (16, 48) | 0.078^‡^ |
| Sirolimus treatment | 124 (55) | 47 (44) | 77 (65) | 0.002^§^ |
| Death | 10 (4) | 9 (8) | 1 (1) | 0.007^§^ |

6MWD, six-minute walk distance; 6MWT, 6-minute walk test; retroperitoneal LAMs, retroperitoneal lymphangioleiomyomas; AMLs, angiomyolipomas; TSC, tuberous sclerosis complex; VEGF-D, vascular endothelial growth factor D; SGRQ, St. George’s Respiratory Questionnaire. ‡. Wilcoxon rank-sum test; §. Chi-square test; *. Fisher's exact test. Data are presented as No. (%) or median (interquartile range).

sTable 2 Multivariable generalized linear regression analysed of factors associated with annual decrease of FEV_1_

| Variables | Estimate | 95% CI | *P* |
| --- | --- | --- | --- |
| Intercept | 188.6 | 90.91 to 286.3 | 0.0002 |
| 6MWD<425.5m | -34.90 | -70.77 to 0.97 | 0.0578 |
| VEGF-D≥800pg/ml | -21.68 | -61.07 to 17.71 | 0.2820 |
| CT severity grade III | -78.41 | -112.1 to -44.72 | ＜0.0001 |
| FEV_1_%pred<70% | -1.242 | -1.944 to -0.540 | 0.0006 |
| Sirolimus treatment | 59.02 | 31.44 to 86.60 | ＜0.0001 |

6MWD, six-minute walk distance; VEGF-D, vascular endothelial growth factor D.
